# Severe disseminated *Talaromyces marneffei* infection in idiopathic CD4 lymphopenia

**DOI:** 10.1016/j.idcr.2025.e02148

**Published:** 2025-01-06

**Authors:** Bingkun Li, Tiantian Li, Qian Lu, Dong Liang, Cunwei Cao

**Affiliations:** a Department of Dermatology and Venereology, The First Affiliated Hospital of Guangxi Medical University, Nanning, Guangxi 530021, China; b Guangxi Key Laboratory of Mycosis Prevention and Treatment, Nanning, Guangxi 530021, China

**Keywords:** Idiopathic CD4 lymphopenia, *Talaromyces marneffei*

## Abstract

Idiopathic CD4 lymphopenia (ICL) is a rare non–HIV-related syndrome, characterized by a reduced CD4 T-cell count and a predisposition to various opportunistic infections. However, *Talaromyces marneffei* (TM) infection has rarely been reported in ICL patients. Here, we report a previously healthy 48-year-old male patient who presented with fever, headache, fatigue, vomiting, and poor appetite. Mycological cultures from blood, bone marrow, liver and spleen were positive for TM. The immunodeficiency evaluation revealed a CD4 T-lymphocyte count of 32 cells/μL, with a negative HIV test. After receiving co-treatment with amphotericin B and voriconazole, the patient showed clinical improvement. At 1-year follow-up, the CD4 T-cell count remained decreased despite the complete resolution of symptoms. The appearance of disseminated TM infection in non-HIV patients should prompt an investigation for the possibility of ICL, as the clinical manifestations can be severe.

## Introduction

Idiopathic CD4 lymphopenia (ICL) is a rare syndrome defined as a CD4 T-lymphocyte count of less than 300 cells/μL or less than 20 % of total T-cells on more than one occasion in the absence of HIV infection and other secondary causes associated with depressed levels of CD4 T-cell [1]. Patients with ICL typically present with opportunistic infections, malignancies, or autoimmune disorders. The most opportunistic infections include cryptococcosis, followed by M. tuberculosis and herpes zoster infections [2].

Talaromycosis, a potentially fatal fungal infection caused by *Talaromyces marneffei* (TM), is endemic in Southeast Asia and Southern China, and primarily occurs in immunocompromised individuals, such as those with HIV infection, adult-onset immunodeficiency symdrome due to anti-interferon-γ autoantibodies, immunosuppressant or steroid therapy, malignant tumors [3], [4]. Here, we reported a case of disseminated TM infection in a 48-year-old male patient with ICL.

## Case reports

A previously healthy man in his 40 s was referred for a 15-day history of fever, headache, fatigue, vomiting, poor appetite. He had been treated with antibiotics 10 days prior, but without any improvement in his symptoms. Upon admission, vital signs included a body temperature of 38.9°C, respiration of 20 breaths/min, heart rate at 116 beats/min, blood pressure of 130/82 mmHg, and O^2^ saturation of 98 % on ambient air. Physical examination revealed mild jaundice of the sclera and skin, along with significant enlargement of the liver and spleen.

**Fig. 1:**
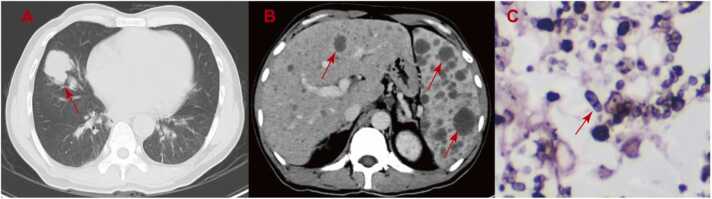
**A** The lung CT showed an irregular mass in the lateral segment of the right middle lobe of the lung with a size of 3.7 × 3.0 × 3.2 cm (arrow); **B** The CT of liver, spleen revealed enlargement of the liver and spleen with multiple low-density lesions (arrow); **C** Wright's stain of the lung mass showed sausage-like shapes yeast organisms with midline septum (arrow, original magnification × 40).

Laboratory tests results are as follows: white blood cell count of 6.87 × 10^9^ /L (normal, 3.5–9.5 ×10^9^ /L), with neutrophils 93 % (40–75 %), total bilirubin 33.9 μmol/L (0–21 μmol/L), direct bilirubin 28.7 μmol/L (0–6.8 μmol/L), alaninetransaminase 132 U/L (13–40 U/L), aspartate aminotransferase 227 U/L (7–45 U/L), high-sensitive c-reactive protein 110.2 mg/L (0–5 mg/L) and erythrocyte sedimentation rate 19 mm/h (0–20 mm/h). A computed tomography (CT) scan revealed an irregular mass in the lateral segment of the right middle lobe of the lung with a size of 3.7 × 3.0 × 3.2 cm, and enlargement of the liver and spleen with multiple low-density lesions (Fig. 1**A, B**). Therefore lung, liver and spleen punctures were performed. The lung puncture revealed sausage-like shapes yeast organisms with midline septum (Fig. 1**C**). Mycological cultures from blood, bone marrow, liver and spleen were positive for TM.

However, Thorough investigations showed no evidence of HIV infection, autoimmune disease, the use of immunosuppressive drugs, and malignancies. Further immunodeficiency evaluation was conducted. Serum immunoglobulin levels showed normal IgG level of 9.61 g/L (8.6–17.4 g/L), IgA level of 1.2 g/L (1–4.2 g/L), IgM level of 0.58 g/L (0.3–2.2 g/L), and elevated IgE level of 318.4 IU/ml (0–100 IU/ml). Complement levels were decreased, with C3 at 0.52 (0.7–1.4 mg/dL) and C4 0.09 (0.1–0.4 mg/dL). Flow cytometry assay revealed reduced CD4 T-cell at 12.66 % (30–46 %) with a count of 32 (561–1137), CD8 T-cell at 20 % (19.2–33.6 %) with a count of 50 (220–1030), B cell at 3 % (8.5–14.5 %) and NK cell at 2.14 % (9.5–23.5 %). Serum anti-interferon-γ autoantibodies testing was negative. Thus, a diagnosis of disseminated TM infection due to ICL was established.

Initially, after receiving intravenous voriconazole (0.2g bid) treatment for 3 days, the patient still had persistent fever. It was then changed to a combination intravenous voriconazole (0.2g bid) and amphotericin B (30mg qd), leading to significant disease remission. After 6 months of oral voriconazole (0.2g bid) therapy, the treatment was changed to oral itraconazole (0.2g bid) owing to dramatic reduction in size of right lung mass, liver and spleen abscess. Lymphocytes was monitored and the results showed low CD4 T-cell, CD8 T-cell, B cell, NK cell counts after 12 months (Table 1). As of December 22, 2024, the patient has been on prolonged itraconazole (0.2g bid) therapy for 20 months.

**Table 1:** Lymphocyte and lymphocyte subset count.

Date of analysis	Counts （/μL）				Percentage （%）			
	CD4 T-cell	CD8 T-cell	B cell	NK cell	CD4 T-cell	CD8-T cell	B cell	NK cell
Before treatment	32	50	NA	NA	12.66	20	3	2.14
Treatment after 4 month	5	384	46	39	0.22	17.68	2.11	1.76
Treatment after 12 month	5	128	14	21	0.67	15.55	1.78	2.67

Normal range: CD4 T-cell counts (561–1137), CD 8 T-cell (220–1030), B cell (180–324), NK cell (200–567); CD4 T-cell percentage (30–46 %), CD8 T-cell percentage (19.2–33.6 %), B cell percentage (8.5–14.5 %), NK cell percentage (9.5–23.5 %). NA: No detection.

## Discussion

The incidence of TM infections in non-HIV infected patients has been increasing in recent year. Anti-interferon-γ autoantibodies, associated with adult-onset immunodeficiency syndrome, are a major cause of disseminated TM infection in non-HIV infected patients [5]. To date, there has been only one case report of cutaneous TM infection in a 38-year-old male patient with ICL [6]. However, this patient did not test anti-interferon-γ autoantibodies. Interestingly, some patients with anti-interferon-γ autoantibodies may develop lymphocytopenia [7]. Therefore, according to the ICL diagnosis standard, a diagnosis cannot be confirmed unless anti-interferon-γ autoantibodies-association immunodeficiency is ruled out. To the best of our knowledge, we are the first to report a case of severe disseminated TM infection in a non-HIV patient without a known immunodeficiency, who was ultimately diagnosed with ICL.

In a retrospective cohort, it was found that comparing to HIV-infected patients, non-HIV-infected patients were significantly older, less likely to have fever, splenomegaly and umbilicated skin lesions, and more likely to have Sweet’s syndrome and osteoarticular lesions. Non-HIV-infected patients also had higher leukocyte, CD4 T-cells and platelet counts, and lower alanine transaminase level and blood culture-positive rate [8]. Despite the absence of evidence for HIV infection, our patient suffered from disseminated TM infection involvling blood, liver, spleen and lung, which seems to be similar to the presentations observed in HIV patients with low CD4 T-cells counts [8], [9]. Therefore, disseminated TM infection in HIV-negative patients should lead to an lymphocyte investigation for the possibility of ICL.

The management of ICL patients is aimed at treating and/or preventing opportunistic infections and increasing the number of CD4 T-cells [10]. There are no established guidelines for disseminated TM infection in ICL patients. There has been only one reported case of TM infection in a patient with ICL, and this patient was cured after treatment with itraconazole [6]. In our patient, the severe manifestations appeared to be significant, and he initially failed to respond to voriconazole treatment. However, his contidions improved after combination with amphotericin B treatment, suggesting more potent antifungal treatment is necessary in cases with severe manifestations. Treatment with different cytokines to raise CD4 T-cells counts, such as IL-2, IFNγ, shows promise [11], [12], [13]. Allogeneic bone marrow transplantation has also be performed in a patient [14]. However, the clinical experience with such treatments is limited. A prospective study indicated that most patients with ICL have persistent low CD4 T-cells counts, and only a small fraction of patients with ICL returned to normal within 3 years of diagnosis [1]. Therefore, it is suggested to follow ICL patients more closely during the first 3 years after diagnosis in order to find out spontaneous recovery, which may help clinicians decide when to discontinue treatment and prophylaxis. Consistent with these findings, and despite symptom improvement, our patient’s lymphocyte counts remained lower than initial levels at the 1-year follow-up without additional treatment to increase lymphocyte counts. Therefore, we have treated our patient with prolonged itraconazole maintenance therapy due to persistently low lymphocyte counts.

In conclusion, we described the clinical features, treatment and prognosis of an ICL patient with TM infection. These case highlights that the appearance of disseminated TM infection in non-HIV patients should prompt an investigation for the possibility of ICL, as the clinical manifestations can be severe.

## Ethical approval

This study has been approved by the Human Ethics Committee of the Affiliated Hospital of Guangxi medical university (Approval No: 2021-KY-138).

## Author Agreement

We confirm that this work is original, we have all seen and approved the final version. This has not been previously published elsewhere, and is not under consideration by another journal or book. We will also not submit the material to another journal until the completion of the editorial decision process at ID Cases. We have no conflicts of interest to disclose. We approve the manuscript and have contributed significantly to the work.

## Disclosures

None.

## Consent

This case report does not contain any identifiable information. Written informed consent was obtained from the patient.

## Funding

This work was supported by the Natural Science Foundation of Guangxi Province of China (AB24010134), and the National Key Research and Development Program of China (2022YFC2504800).

## CRediT authorship contribution statement

**Dong Liang:** Formal analysis, Software. **Qian Lu:** Formal analysis, Software. **Tiantian Li:** Investigation, Writing – original draft. **Bing kun Li:** Conceptualization, Data curation, Writing – original draft. **Cunwei Cao:** Supervision, Writing – review & editing.

## Declaration of Competing Interest

The authors declare that they have no known competing financial interests or personal relationships that could have appeared to influence the work reported in this paper.
